# Long-term muscarinic inhibition increases intrinsic excitability through the upregulation of A-type potassium currents in cortical neurons

**DOI:** 10.3389/fcell.2025.1570424

**Published:** 2025-05-27

**Authors:** Denise Riquelme, Patricia Romo-Toledo, Paula Leyton, Claudio Moreno, Elias Leiva-Salcedo

**Affiliations:** Departmento de Biología, Facultad de Química y Biología, Universidad de Santiago de Chile, Santiago, Chile

**Keywords:** intrinsic excitability, A-type potassium currents, cortical neurons, homeostatic regulation of excitability, muscarinic acetylcholine receptor (mAChR), nicotinic acetylcholine receptor (nAChR)

## Abstract

Neurons undergo a series of perturbations that alter their firing rate and synaptic transmission; however, they can adapt to keep a target level of electrical activity in the long term. Muscarinic receptor (mAChR) transmission modulates intrinsic excitability and allows for fast changes through phasic transmission and long-term effects through volume transmission. Earlier studies on mAChR transmission have primarily focused on the effects of long-term mAChR stimulation on excitability; however, the impact of long-term inhibition is still unknown. In this study, we used a combination of patch-clamp and immunofluorescence techniques to examine the effects short-term (3 h) and long-term (0–10 days) muscarinic or nicotinic (nAChR) receptor inhibition on the intrinsic excitability of cortical pyramidal neurons in culture. We found that short term mAChR or nAChR inhibition has no effect either in AIS or in neuronal excitability, however, prolonged mAChR, but not nAChR blockade, increases the AIS length with no change in its position. Moreover, prolonged mAChR blockade increases firing frequency and intrinsic excitability, through a reduction in the action potential duration that is the result of an increase in a 4-AP sensitivity K^+^ current in cortical pyramidal neurons in culture. Together, our work demonstrates that prolonged mAChR, but not nAChR, blockade induces structural and functional changes to compensate for the lack of mAChR signaling and to sustain a target level of electrical activity.

## 1 Introduction

Neuronal activity is shaped by both synaptic transmission and intrinsic excitability. Changes in either of these functions affect neuronal dynamics and circuit excitability. To maintain long-term stability despite fluctuations in network activity or excitability, while still retaining short-term flexibility, neurons adapt through modifications in their synaptic architecture, function, and/or intrinsic excitability (Turrigiano, 2011). Neurotransmitters and neuromodulators play a crucial role in these mechanisms. Recent studies have demonstrated homeostatic changes during extended periods of cholinergic stimulation (Puhl et al., 2023). The influence of cholinergic signaling on neurons depends on the specific receptor activated, and the absence of this mechanism may destabilize circuit activity.

Cholinergic activity affects neurons through two primary mechanisms: calcium influx—mediated by nicotinic receptors and M1, M3, and M5 G_q_-protein-coupled muscarinic receptors, and cAMP modulation via M2 and M4 G_i_/G_o_-protein-coupled receptors (Lucas-Meunier et al., 2003; Obermayer et al., 2017). Long-term activation of cholinergic receptors (48 h) increases the number of neurons exhibiting hyperadapting behavior by upregulating the M current (Kv7), thereby reducing intrinsic excitability. However, this effect is rapidly reversed by acute cholinergic stimulation (Puhl et al., 2023). Conversely, prolonged inhibition of the M current through cholinergic stimulation or Kv7 inhibition decreases excitability by relocating Kv7 and Nav channels to the distal part of the axon initial segment (AIS), shifting the action potential initiation zone in a CK2-dependent manner (Lezmy et al., 2017). The effects on intrinsic excitability become evident after 48 h, while structural changes are observed within 1 h of treatment (Lezmy et al., 2017). The interaction between long-term cholinergic signaling and intrinsic excitability contributes to stabilizing neuronal responses, establishing a new homeostatic state.

Structural changes in the AIS play a critical role in action potential generation and the regulation of synaptic transmission. Long-term auditory deprivation elongates the AIS and increases neuronal excitability, an effect mediated by a decrease in Kv1.1 and an increase in Kv7.2 in the AIS (Kuba et al., 2010; Kuba et al., 2015). Conversely, continuous depolarization with KCl for 48 h reduces AIS length and shifts the AIS away from the soma, thereby decreasing excitability (Grubb and Burrone, 2010a). These AIS modifications regulate neuronal excitability, compensating for reduced activity by increasing intrinsic excitability (Wise et al., 2024a; Wise et al., 2024b) or, in the case of sustained depolarization, reducing neuronal excitability. Together, these findings indicate that neurons undergo structural adaptations to counteract excessive or insufficient activity, thereby maintaining a target level of excitability.

While the homeostatic regulation of excitatory synaptic transmission and intrinsic excitability has been extensively studied (Kuba et al., 2010; Nataraj et al., 2010; Turrigiano, 2012; Campanac and Hoffman, 2013) the effects of short- and long-term cholinergic blockade remain unexplored. To address this, we used a combined strategy using immunofluorescence and electrophysiological recordings in cortical neuron cultures to examine the impact of prolonged cholinergic inhibition on AIS structure and intrinsic excitability. Our findings demonstrate that long-term mAChR inhibition elongates the AIS and increases firing frequency via upregulation of 4-AP-sensitive currents. This effect is specific to long-term mAChR inhibition, as nAChR inhibition, whether short- or long-term, has no effect on excitability or AIS structure. Overall, our results suggest that prolonged mAChR inhibition induces a homeostatic adaptation in pyramidal neurons, compensating for the absence of mAChR signaling.

## 2 Methods

All experiments were conducted in accordance with animal protocols approved by the Ethics Committee of the Universidad de Santiago de Chile (N° 692/2018) and followed the guidelines of the National Research and Development Agency (ANID). Male and female C57BL/6J mice were housed in a temperature- and humidity-controlled facility with a 12/12-h light/dark cycle and provided with water and food *ad libitum*.

### 2.1 Mouse cortical neuron culture

Plating medium (MEM supplemented with 20% horse serum, 0.1% glucose, 0.5 mM sodium pyruvate, 10 mM HEPES, and 100 IU/mL penicillin-streptomycin) and neuronal culture medium (Neurobasal medium supplemented with 2% B27, 1% GlutaMAX-I, and 100 IU/mL penicillin-streptomycin) were prepared on the day of culture and stored at 4°C for no longer than 7 days.

Primary cortical neurons were isolated from E18 C57BL/6J mouse embryos. The cortices were dissected in Hank’s Balanced Salt Solution (HBSS) containing 5 mM MgCl_2_ (pH 7.4). After meninges removal, the cortices were minced with tweezers and transferred to a 15 mL tube, washed twice with HBSS, and digested with 0.25% trypsin and 0.03 mg/mL DNase I for 8 min at 37°C. The trypsin solution was removed by washing three times with plating medium. The tissue was then triturated eight times using a fire-polished Pasteur pipette, and the resulting suspension was passed through a 40 μm cell strainer. Cells were counted using a hemocytometer and seeded at a density of 40,000 cells per well on 12 mm coverslips precoated with 30 μg/mL poly-D-lysine and 2 μg/mL laminin. After 4 h, the plating medium was replaced with Neurobasal culture medium. Neurons were cultured at 37°C with 5% CO_2_, and one-third of the neuronal culture medium was replaced every 3 days.

### 2.2 Cell treatments

For long-term treatment, 10 µM atropine or 10 µM mecamylamine was added to cortical neuron cultures from DIV 0 to 10, with the drugs replenished at each medium change. For short-term treatment, neurons at DIV 10 were incubated with 10 µM atropine or 10 µM mecamylamine for 3 h at 37°C in 5% CO_2_. All treatments were performed at DIV 10, and neurons were subsequently analyzed via electrophysiology and immunolabeling. Drug solutions were prepared according to the manufacturer’s instructions and stored as frozen stock solutions.

### 2.3 Primary and secondary antibodies

The following primary antibodies were used: monoclonal anti-ankyrin G (Anti-AnkG) (75–146, RRID: AB_10673030) and monoclonal anti-PSD-95 (75-028, RRID: AB_2292909) from NeuroMab (United States); polyclonal anti-MAP2 (ab32454, RRID: AB_776174) from Abcam (United States); and polyclonal anti-Neurogranin (AB5620, RRID: AB_91937) from EMD Millipore (United States). Secondary antibodies included Alexa Fluor 488 goat anti-mouse IgG2a (A-21131), Alexa Fluor 488 goat anti-mouse IgG1 (A-21121), and Alexa Fluor 546 donkey anti-rabbit (A10040) from Life Technologies (United States).

### 2.4 Immunofluorescence labeling

Neurons were fixed in 4% (w/v) formaldehyde in phosphate-buffered saline (PBS, pH 7.4) for 15 min at room temperature and washed three times with PBS. Cells were then permeabilized with 0.25% Triton X-100 for 5 min and blocked for 1 h in either 10% goat serum (for AnkG, Neurogranin, or MAP2) or 5% goat serum with 1% BSA and 0.3% Triton X-100 (for PSD-95). Primary antibodies were diluted in 10% goat serum and incubated overnight at 4°C at the following concentrations: AnkG (1:100), Neurogranin (1:300), MAP2 (1:300), and PSD-95 (1:100). After three PBS washes, cells were incubated with secondary antibodies for 1.5 h at room temperature, washed three times with PBS, and mounted in Prolong Gold Antifade Mounting Medium.

### 2.5 Acquisition and analysis of images

Images were acquired using a Zeiss LSM 800 laser-scanning confocal microscope equipped with appropriate excitation and emission filters, a ×40 oil immersion objective (1.4 NA), and a pinhole set to 1 AU. Laser power and gain settings were adjusted to prevent signal saturation. Z-stacks were acquired with 0.5 µm step sizes using a 3× zoom (for AIS measurement) or without zoom (for PSD-95 quantification). Acquisition parameters were kept constant across experiments.

For AIS measurements, Z-stacks were converted into maximum intensity projections using Fiji software and analyzed with a MATLAB script (www.mathworks.com/matlabcentral/fileexchange/28181-ais-quantification) (Grubb and Burrone, 2010a). The AIS start and end positions were determined based on Ankyrin G staining intensity, and length was measured accordingly. PSD-95 quantification was performed using Fiji ImageJ with NeuronJ and SynapCountJ plugins (https://spineup.jimdofree.com/downloads/).

### 2.6 Electrophysiological recordings

Coverslips containing cortical neurons in culture at DIV 10 were placed on a stage of a Nikon Ti2 microscope and continuously perfused with oxygenated Krebs-like buffer containing (in mM): 145 NaCl, 5 KCl, 1 MgCl_2_, 2.5 CaCl_2_, 10 HEPES, 10 glucose, pH 7.4, 300 mOsm/kg of osmolality, all experiments were performed at room temperature. Whole cell recordings were made in cultured cortical neurons using borosilicate glass pipettes (4–6 MΩ) filled with intracellular solution. For voltage and current clamp, the intracellular solution contained (in mM): 120 potassium-gluconate, 10 KCl, 8 NaCl, 10 HEPES, 0.5 EGTA, 4 Mg-ATP, 0.3 Na-GTP, pH 7.2 adjusted with KOH (∼300 mOsm/kg). For voltage clamp recordings, the pipette and whole cell capacitance were compensated, series resistance was compensated by 80%, neurons were held at −80 mV and recorded in Krebs-like buffer. The voltage-step protocols consisted in voltage change from −100 to 50 mV with 10 mV steps from a holding potential of −80 mV, with a duration of 0.5 s and delivered somatically at 0.2 Hz. Liquid junction potential was 16.8 mV and was not subtracted (calculated according to the stationary Nernst–Planck equation (Marino et al., 2014) using LJPcalc software (https://swharden.com/LJPcalc). The current-step protocols consisted in a series of somatic current injections from −200 to 400 pA in steps of 50 pA with a duration of 1 s and delivered at 0.2 Hz. Current-ramp protocol consisted in a continuous current change at a rate of 4 pA/ms from −200 to 600 pA, from a holding current of 0 pA. Capacitance neutralization was applied and recording showing changes in access resistance >20% were discarded from the analysis. Voltage and current clamp recordings were performed using an Axopatch 200B (Molecular Devices, United States) and HEKA EPC-10 (HEKA Electronic, Germany), and digitized using a National Instruments PCIE-6323 (NI, United States), data was low-pass filtered at 10 kHz and digitized at 50 kHz using WinWCP 5.6 (https://github.com/johndempster/WinWCPXE/releases/tag/V5.7.8). The voltage-dependent activation curve of Kv currents were fitted by a Boltzmann equation as follows; 1/(1+exp[zF(V_0.5_-V)ß]).

### 2.7 Data analysis

Electrophysiological data were analyzed with Python 3.7 using the eFEL module (https://github.com/BlueBrain/eFEL). Rheobase was defined as the minimal current step to trigger an action potential. The input resistance and time constant were monitored using a hyperpolarizing pulse of 50 pA for 10 ms at the end of the current step. The AP duration was measured as full width at half maximum amplitude from the onset. Onset was defined as the change in the voltage slope >10 mV/ms. The AP amplitude was measured from the membrane potential to the peak amplitude. The threshold was defined as the voltage at which the slope reaches 20 mV/ms. Rise time was measured between 20%–80% of the time between the onset and the peak amplitude. Decay time was measured at 37% of the decaying phase from the peak amplitude. Latency was measured from the onset of the current injection to the onset of the first AP. Adaptation index was defined as the normalized average difference of two consecutive interspike intervals, skipping the first two intervals. Post-spike voltage was measured from the AP threshold to the minimum voltage after the peak amplitude.

EPSP and EPSC measurements were performed for at least 2 min, frequency and amplitude was measured using a template matching function (Clampfit 10, Molecular Devices, United States) to skip AP firing events during the recording. The frequency and amplitude of the AP were not counted in the EPSPs frequency and amplitude analysis.

Data are reported as mean ± standard deviation in text and in most of the graphs as a box plot showing the standard deviation, the median, the 95% confidence interval (CI) and the density plots. Data was tested for normal distribution using the Shapiro–Wilk test. For parametric data, statistical significance between groups means was assessed using one-way ANOVA followed by a Tukey multiple comparisons *post hoc* test. For grouped analysis of current/voltage and firing frequency/current experiments, we used two-way ANOVA followed by a Šídák *post hoc*. For a two-group comparison, we used the *t*-student test, non-parametric data were tested with the Wilcoxon rank test. Statistical significance was determined at *p* < 0.05. All statistical tests were performed using JASP software (https://jasp-stats.org/), and the curve fitting was performed using SciDavis 2.8 (https://scidavis.sourceforge.net/).

## 3 Results

### 3.1 Prolonged mAChR blockade increases AIS length

To determine the impact of cholinergic blockade on AIS structure, we performed immunofluorescence labeling for Ankyrin G, a well-established AIS marker, and quantified the length and position of the AIS in cultured cortical neurons at DIV 10. Two experimental conditions were used: (1) short-term (3 h) muscarinic or nicotinic receptor inhibition to assess the effects on a fully formed AIS, and (2) long-term (0–10 DIV) cholinergic receptor inhibition to examine the impact of inhibition before and during AIS formation.

**FIGURE 1:**
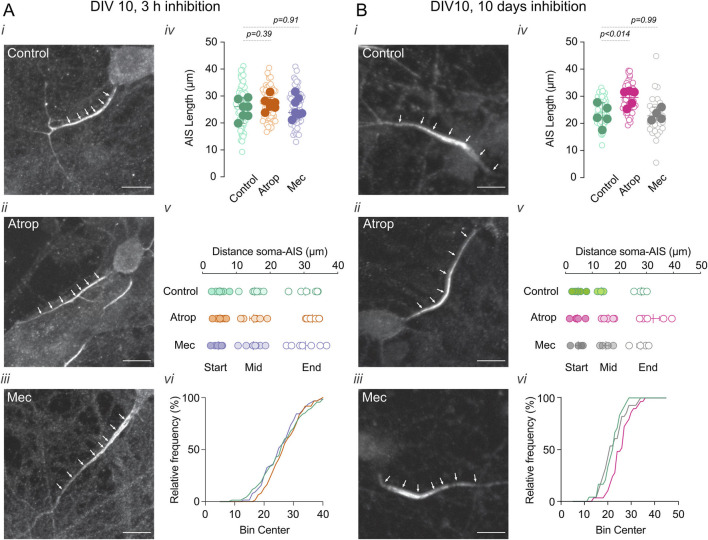
Chronic inhibition of muscarinic transmission increases AIS length. Effect of 3 h **(A)** and 0–10 DIV **(B)** incubation with cholinergic receptor inhibitors atropine or mecamylamine in the AIS length and position. Neurons were immunolabeled for Ankyrin G (white arrowheads), a marker for the AIS. In each panel **(A,B)** (*i*) shows a representative image of neurons in control, (*ii*) 10 µM atropine, and (*iii*) 10 µM mecamylamine treatment. (*iv*) Shows the summary plots of AIS length with the different treatments (open circle indicates individual cells, closed circles indicate the average of the cells on independent cultures), (*v*) shows the summary plot of the distance of the soma-AIS, s, start; M, middle; E, end (circles represent the average of cells in each culture). (*vi*) Shows a representative frequency distribution of the AIS intensity signal. Scale bar = 10 µm. Statistical differences were tested using an unpaired Student’s *t*-test, *p*-values are showed above the graphs.

We found that a 3-h cholinergic receptor inhibition had no effect on AIS length or position (Figure 1A). In contrast, long-term inhibition of muscarinic acetylcholine receptors (mAChRs) with 10 µM atropine increased AIS length (Figure 1B) without altering its position. Conversely, long-term nicotinic acetylcholine receptor (nAChR) inhibition with 10 µM mecamylamine had no effect on AIS length or position (Figure 1B). Together, our findings suggest that neurons compensate for prolonged reductions in mAChR activity by enlarging their AIS.

### 3.2 Prolonged mAChR inhibition increases intrinsic excitability and reduced AP duration

**FIGURE 2:**
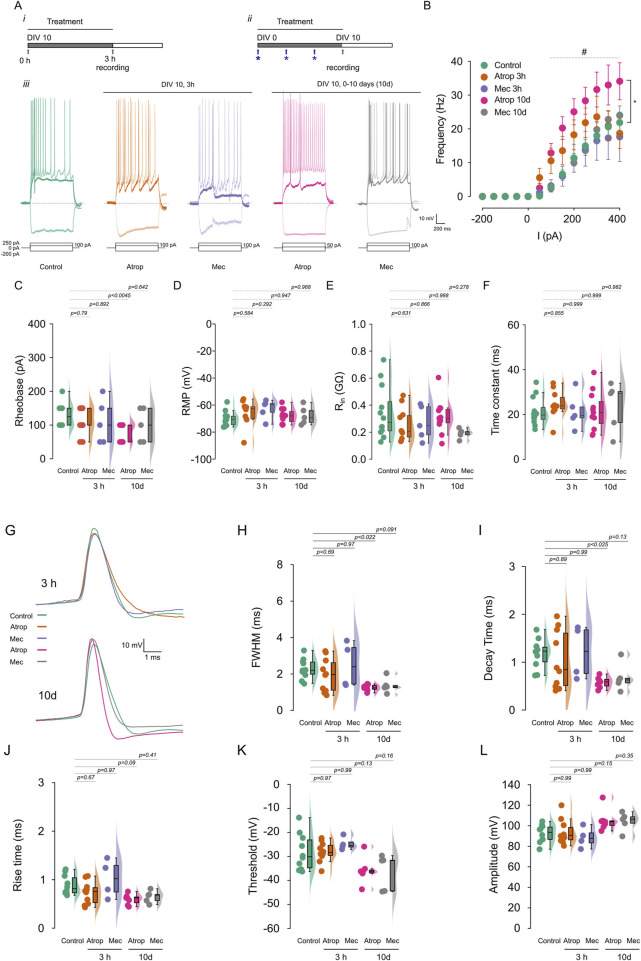
Chronic muscarinic blockade increases firing frequency. Cortical neurons in culture were treated with 10 µM atropine or 10 µM mecamylamine for 3 h or 0–10 DIV, and the firing frequency was measured using current clamp. **(A)** Show the experimental protocols for 3 h (i) and 0–10 DIV treatments (ii) and the representative voltage traces (iii) recorded at −200 pA, rheobase and 250 pA of current injection. **(B)** Shows the summary graph of the AP firing frequency. **(C)** Show the rheobase, **(D)** the RMP, **(E)** the R_in_ and **(F)** the time constant of cortical pyramidal neurons measured before the current-step protocols. **(G)** AP waveform of the first action potential at rheobase, extracted from the current-step protocols and analyzed in the different treatment. **(H)** Shows the FWHM, **(I)** the decay time, **(J)** the rise time, **(K)** the threshold, and **(L)** the amplitude of AP. Statistical differences for **(B)** were tested using two-way ANOVA, with a Šídák *post hoc* test. All other analysis were conducted using one-way ANOVA, with a Tukey *post hoc* test, *p*-values are showed above each graph. Star indicates the comparison between control and atropine 0–10 DIV treatment and # indicates *p* < 0.05 in this comparison.

Activity-dependent changes in AIS structure can influence intrinsic neuronal excitability (Kuba et al., 2010). To investigate the effects of cholinergic receptor blockade on neuronal excitability, we performed current-clamp recordings in cultured cortical neurons treated with either 10 µM atropine or 10 µM mecamylamine for 3 h or from 0 to 10 DIV (Figures 2Ai,ii). We found that only the 0–10 DIV atropine treatment increased firing frequency, with this effect appearing at somatic current injections from 100 pA to 400 pA (Figures 2Aiii,B), and reduced the rheobase by approximately 40% (Figure 2C; Supplementary Figures S1A,B). Analysis of passive properties (Supplementary Table S1) revealed that cholinergic receptor inhibition, whether for 3 h or from 0 to 10 DIV, had no effect on resting membrane potential (RMP) (Figure 2D), input resistance (R_in_) (Figure 2E), or membrane time constant (Figure 2F). Additionally, we observed no change in latency but a reduction in the interspike interval (Supplementary Figures S1A,C). Our results demonstrate that long-term mAChR blockade enhances intrinsic neuronal excitability and suggests a compensatory mechanism leading to a new excitability state.

**FIGURE 3:**
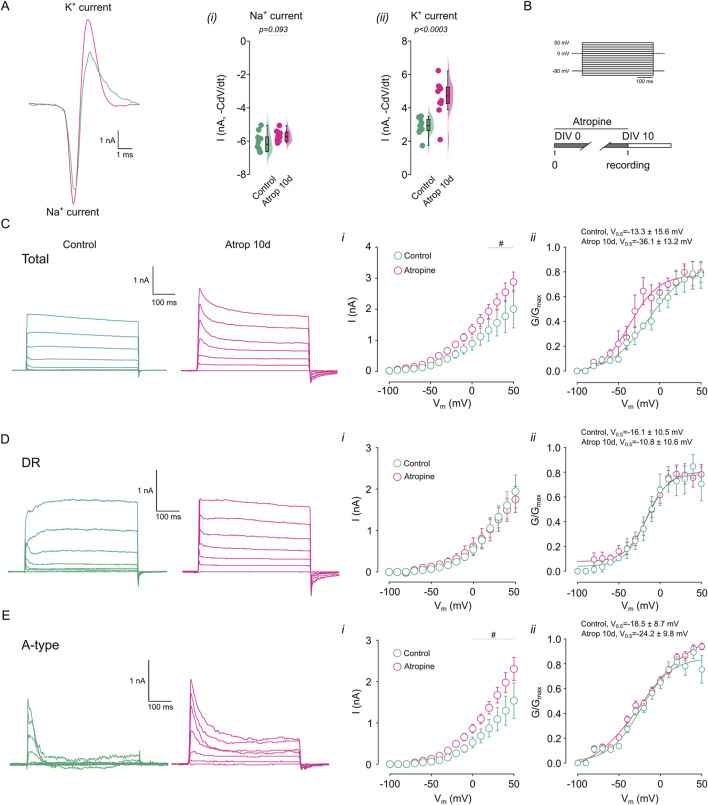
Chronic muscarinic inhibition increases K^+^ currents. The net ion current was calculated from the action potential waveform in cortical pyramidal neurons treated for 0–10 DIV with 10 µM atropine. **(A)** Shows the calculated ionic current underlying the action potential in the different treatments, right panels show the quantification of the (i) Na^+^ and (ii) K^+^ current. **(B)** Shows the voltage step protocol used for the current measurement. **(C)** Shows the representative traces of the total K^+^ current obtained in the presence of 0.5 µM TTx and measured in control and atropine treated neurons, right panels show the (i) I/V plot, (ii) the G/G_max_ curve. **(D)** Shows the representative traces of the DR K^+^ current obtained in the presence of 0.5 µM TTx and 2 mM 4-AP and measured in control and atropine treated neurons, right panels show the (i) I/V plot, (ii) the G/G_max_ curve. **(E)** Shows the representative traces of the 4-AP sensitive K^+^ current obtained by subtracting total K^+^ currents for DR K^+^ currents and measured in control and atropine treated neurons, right panels show the (i) I/V plot, (ii) the G/G_max_ curve. Statistical differences for I/V plots were tested using two-way ANOVA, with a Šídák *post hoc* test. All other comparisons were tested using an unpaired Student’s *t*-test, *p*-values are showed above the graph. # indicates voltage were *p* < 0.05 in this comparison.

To investigate the effects of cholinergic receptor blockade on action potential (AP) waveforms, we analyzed the first AP evoked at the rheobase. We observed that 3-h treatments with atropine or mecamylamine had no effect on the AP waveform (Figures 2G–L). However, prolonged 0–10 DIV atropine treatment resulted in a shorter AP duration, characterized by a more negative post-spike voltage (Figures 2G,H; Supplementary Table S1). This reduction in AP duration was primarily due to a decrease in decay time (Figures 2G,I), without affecting rise time (Figures 2G,J), AP threshold (Figures 2G,K), or amplitude (Figures 3G,L). In contrast, 0–10 DIV mecamylamine treatment had no effect on the AP waveform. These findings suggest an upregulation of potassium currents involved in AP repolarization. Moreover, our results indicate that long-term mAChR blockade enhances neuronal excitability through a mechanism involving ion channels critical for AP generation.

### 3.3 Prolonged mAChR inhibition increases 4-AP sensitive current

Since we observed effects on intrinsic excitability only with muscarinic receptor blockade, we further examined the consequences of long-term mAChR inhibition. We hypothesized that the reduction in AP duration might result from alterations in Na^+^ and/or K^+^ currents. To test this, we characterized the ionic currents underlying AP generation by calculating the net ion current from the AP waveform using the formula -C × dV/dt, where C represents capacitance and dV/dt denotes the time derivative of voltage (Carter and Bean, 2009). We observed no change in the Na^+^ current (Figure 3Ai), but an increase in the K^+^ current (Figure 3Aii) following 0–10 DIV atropine treatment. To further investigate the role of K^+^ currents in the effects of long-term mAChR inhibition, we recorded K^+^ currents using a voltage-step protocol (Figure 3B, Methods). We found that 0–10 DIV atropine increased total K^+^ currents between 20 and 50 mV (Figure 3Ci), with no change in voltage sensitivity (Figure 3Cii). Next, we isolated the K^+^ current using 2 mM 4-AP, to inhibit A-type K^+^ current and measured the residual current (DR). We found that long-term mAChR blockade did not alter the DR K^+^ current or its voltage sensitivity (Figures 3Di,ii). Conversely, 0–10 DIV atropine increased the 4-AP-sensitive K^+^ current between 0 and 50 mV (calculated by subtracting the DR K^+^ current from the total K^+^ current) (Figure 3Ei), without affecting voltage sensitivity (Figure 3Eii). Collectively, these data demonstrate an upregulation of 4-AP-sensitive K^+^ currents following chronic mAChR blockade, suggesting a role for these currents in the repolarization phase of the action potential and contributing to increased firing frequency.

### 3.4 Increased synaptic activity after chronic deprivation of muscarinic signaling

**FIGURE 4:**
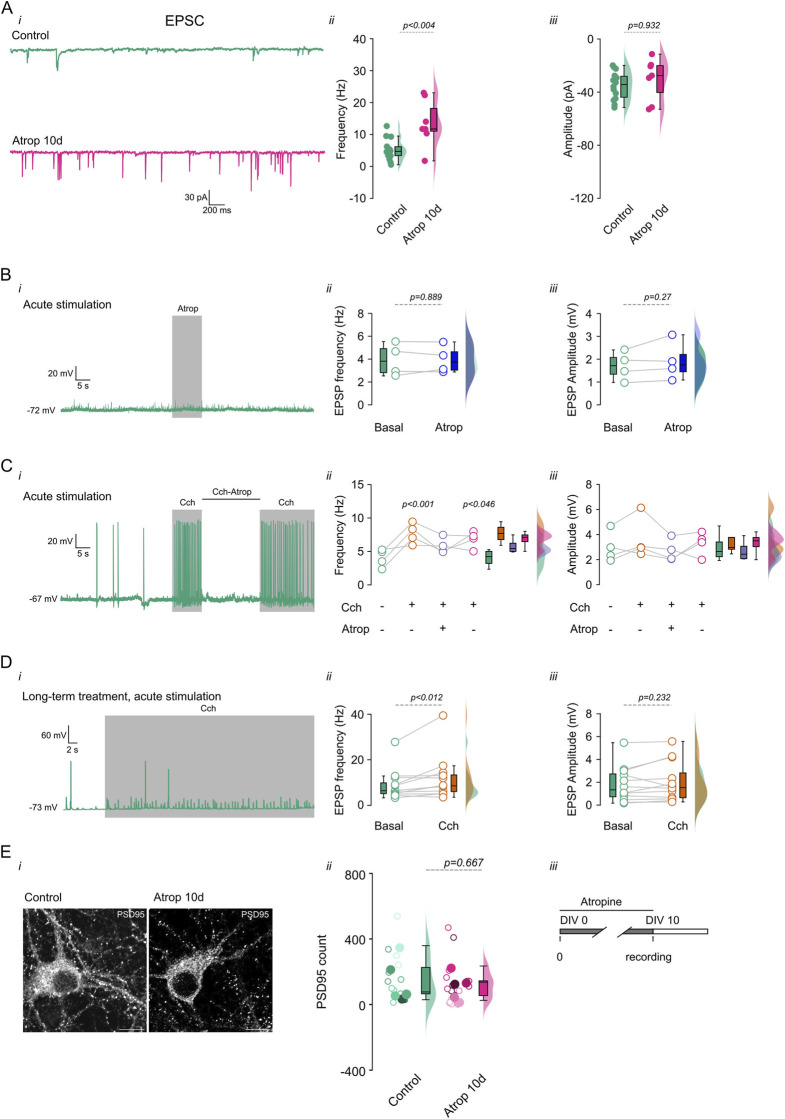
Increased synaptic transmission after long term cholinergic receptor inhibition. Chronic inhibition of muscarinic receptors increases synaptic transmission. Cortical neurons in culture were treated with 10 µM atropine from 0–10 DIV and the synaptic events were measured (sEPSC). (**A**, i) Shows the current traces. (**A**, ii) shows the frequency and (**A**, iii) the amplitude of the sEPSC. (**B**, i) Shows voltage trace showing the effect of 10 s 10 µM atropine perfusion, (**B**, ii) show the quantification of the frequency and (**B**, iii) amplitude of the sEPSP. (**C**, i) Shows the voltage trace showing the effect of acute 10 µM Cch and 10 µM Cch +10 µM atropine, and the right panels show the (**C**, ii) frequency and (**C**, iii) amplitude of the sEPSP. (**D**, i) Shows the voltage traces showing the effect of acute perfusion of 10 µM Cch on 0–10 DIV atropine treated neurons, the right panels show the quantification of the (**D**, ii) frequency and (**D**, iii) amplitude of the sEPSP. (**E**, i) Showed the immunofluorescence for PSD-95 and (**E**, ii) the quantification of the signal at 0–10 DIV atropine incubation, open circles indicate individual cells, filled circles indicates the independent cultures, each color indicates the cells from the respective culture. (**E**, iii) depict the mAChR inhibition protocols. The box plot represents the median and 95% CI, the semi violins show the distribution of the data. Statistical differences were tested using a paired *t*-test (in A, B, and D), unpaired *t*-test (in E) and one-way ANOVA with a Tukey *post hoc* (in C), *p*-values are showed above each graph. Calibration bar = 10 µm.

To investigate the effects of long-term mAChR inhibition on synaptic transmission in cultured cortical neurons, we incubated neurons with 10 µM atropine from 0 to 10 DIV. We found that atropine increased sEPSC frequency (Figures 4Ai,ii; Supplementary Table S2) but not amplitude (Figures 4Ai,iii). Additionally, in non-inhibited neurons, acute 10 µM atropine had no effect on sEPSPs (Figure 4B). However, as previously described (Williams and Kauer, 1997; Gulledge et al., 2009), it blocked the effects of cholinergic transmission (10 µM Cch) (Figure 4C). To determine whether long-term atropine treatment altered neuronal sensitivity to Cch, we recorded sEPSPs in neurons treated with 0–10 DIV atropine and then acutely perfused them with 10 µM Cch. We found an increase in sEPSP frequency (Figures 4Di,ii), with no change in amplitude (Figures 4Di,ii). These results demonstrate that neurons remain responsive to Cch despite long-term muscarinic inhibition, suggesting that mAChRs remain functional.

The increase in frequency of synaptic events after long-term inhibition of muscarinic receptors can be explained by an increase in the glutamatergic inputs onto the pyramidal neurons or an increased vesicle release. To address these, we performed immunostaining for PSD-95, a synaptic marker, and measured puncta in mAChR inhibited pyramidal neurons. We found no change in PSD-95 signal at 0–10 DIV atropine treatment (Figure 4E), showing that chronic mAChR inhibition does not change synaptic inputs onto pyramidal neurons, and suggesting that neurons adapt to long-term muscarinic blockade by increasing neurotransmitter release.

## 4 Discussion

This study demonstrates that long-term muscarinic acetylcholine receptor (mAChR) inhibition induces elongation of the axon initial segment (AIS), increases firing frequency and intrinsic excitability, and upregulates a 4-AP-sensitive potassium current. These effects are specific to prolonged mAChR inhibition and do not occur with short-term inhibition or nicotinic acetylcholine receptor (nAChR) blockade. Our findings highlight a compensatory mechanism that modulates neuronal excitability, enabling activity-dependent regulation of neuronal output.

### 4.1 AIS plasticity after cholinergic receptor inhibition

Our results show that short-term cholinergic receptor blockade, whether nicotinic or muscarinic, has no impact on AIS length or excitability. In contrast, long-term mAChR inhibition, but not nAChR inhibition, leads to AIS elongation and increased neuronal excitability. Notably, short-term mAChR stimulation induces a distal shift of Kv7.3 channels within the AIS, reducing excitability (Lezmy et al., 2017). Additionally, cholinergic stimulation (48 h) slightly shifts the AIS toward the soma without altering its length but induces a hyperadapting firing pattern that reduces firing frequency. This effect is reversible with subsequent cholinergic stimulation (Puhl et al., 2023). These observations suggest a homeostatic mechanism in excitability regulation, contrasting with our findings that long-term mAChR inhibition produces the opposite effects, increasing neuronal excitability by elongating the AIS.

Our experimental approach, which involved long-term pharmacological treatments from 0 to 10 DIV, was designed to observe effects occurring before and during the establishment of the axon initial segment (AIS) (Grubb and Burrone, 2010b). As previously reported, Nav channel concentration peaks after DIV 7 (Yang et al., 2007), and the maturation of the AIS, initiated around DIV 3–4, is largely complete by DIV 10 (Boiko et al., 2007). During this period, several neuronal changes take place, including the establishment of neuronal excitability through the accumulation of ion channels in the AIS and the enrichment of the AIS with AnkG (Grubb and Burrone, 2010b). Furthermore, our results indicate that muscarinic inhibition significantly affects both excitability and structural changes in the AIS during this developmental window. However, once the AIS is fully established, the effect of muscarinic inhibition becomes minimal, suggesting a limited time window for its influence.

The AIS plays a crucial role in action potential (AP) initiation and neuronal output regulation. Structural plasticity of the AIS in response to prolonged changes in excitatory transmission such as sensory deprivation, long-term inhibition using kynurenic acid (which blocks glutamatergic transmission), or cholinergic stimulation has been shown to adjust excitability through changes in Nav and Kv channel distribution (Kuba et al., 2010; Kuba et al., 2015; Leterrier et al., 2015; Zbili et al., 2021; Puhl et al., 2023). Our findings align with this adaptive framework, demonstrating that chronic mAChR inhibition increases AIS length and excitability as a compensatory response to reduced cholinergic signaling.

### 4.2 Ion channel changes after mAChR inhibition

The effects of mAChR inhibition on excitability may arise from changes in potassium currents. While mAChR stimulation reduces resting Kv7.3 currents, leading to depolarization and reduced firing frequency (Lezmy et al., 2017), our experiments indicate that prolonged mAChR inhibition does not affect basal K^+^ currents or membrane resistance, suggesting no impact on subthreshold currents. Instead, the observed increase in excitability correlates with an upregulation of 4-AP-sensitive K^+^ currents, which reduce repolarization time, facilitating faster recovery from Nav channel inactivation (Koyama and Appel, 2006; Khaliq and Bean, 2010), and promoting higher firing rates.

The reduction in AP duration observed in our study may be attributed to increased Kv3 or Kv4 activity, as these channels play a role in AP repolarization (Bean, 2007). While activity-deprived CA3 pyramidal neurons exhibit increased excitability due to Kv1.1 downregulation (Kuba et al., 2010; Kuba et al., 2015; Morgan et al., 2019; Zbili et al., 2021), our results suggest that Kv1.1 is not involved, as we observed no effects on AP latency. Furthermore, Kv4 channels, primarily somatodendritic (Burkhalter et al., 2006) are unlikely contributors to the excitability changes observed, as their role is to stabilize synaptic excitation during dendritic depolarization. Instead, Kv3 channels, which are expressed in the axon and presynaptic terminal (Kaczmarek and Zhang, 2017; Richardson et al., 2022), are likely candidates for mediating fast repolarization and shortening AP duration (Bean, 2007). These channels exhibit classical A-type kinetics and are sensitive to 4-AP (Kaczmarek and Zhang, 2017) However, further experiments are required to fully elucidate their role.

Unexpectedly, we found no effect of nAChR inhibition, whether short- or long-term. While no prior studies have examined the effects of chronic nAChR inhibition, long-term (10-day) exposure to nicotine is known to downregulate nAChR function in neurons from various brain regions (Marks et al., 1993). Moreover, brief (15-min) nicotine stimulation in pedunculopontine neurons enhances spontaneous excitatory synaptic activity (Rowell and Duggan, 1998; Covernton and Lester, 2002). However, prolonged (24-h) nicotine treatment induces long-term receptor inactivation, preventing nicotine from facilitating glutamatergic release (Girod and Role, 2001).

### 4.3 Synaptic transmission

Recordings of excitatory synaptic activity in primary cultures treated with long-term mAChR inhibition revealed a significant increase in synaptic event frequency without changes in amplitude. Similarly, monocular deprivation has been shown to increase both intrinsic excitability and synaptic release probability (Nataraj et al., 2010; Lambo and Turrigiano, 2013), allowing neurons to prioritize burst AP responses (Lisman, 1997; Kepecs et al., 2002; Kepecs and Lisman, 2003). Together, our results suggest that increased intrinsic excitability and enhanced excitatory synaptic transmission serve as homeostatic mechanisms in our study model (Wen and Turrigiano, 2021).

### 4.4 Implications for neuronal function

The upregulation of A-type K^+^ currents enhances neuronal firing by counterbalancing Na^+^ influx and shortening AP duration, the increase in A-type K^+^ currents facilitates faster AP generation and recovery, thereby elevating excitability (Bean, 2009; Kaczmarek and Zhang, 2017). This mechanism aligns with our observations and provides a plausible explanation for the increased firing frequency under prolonged mAChR inhibition. Our findings suggest that AIS elongation and increased excitability under chronic mAChR inhibition represent homeostatic adaptations that stabilize neuronal activity in a highly excitable state. These changes have broad implications for understanding how neurons adjust their output in response to the altered cholinergic tone, contributing to the fine-tuning of neural responses.

While short-term mAChR inhibition did not induce changes in intrinsic excitability or synaptic transmission, long-term inhibition led to increased firing and higher sEPSC frequency. These results fit within the broader framework of homeostatic plasticity, in which postnatal neurons initially compensate through changes in synaptic transmission and intrinsic excitability. After development, neurons regulate excitability by adjusting synaptic scaling and modifying inhibitory transmission (Wen and Turrigiano, 2021). In summary, we found that long-term inhibition of cholinergic transmission enhances excitability to compensate for the lack of muscarinic signaling, highlighting its critical role in postnatal development and its regulation of neuronal output at different developmental stages.

## Data Availability

The raw data supporting the conclusions of this article will be made available by the authors, without undue reservation.

## Supplementary material

SUPPLEMENTARY FIGURE S1 Long term mAChR inhibition reduces rheobase. Effect of long-term mAChR inhibition with 10 µM atropine in the rheobase measured with a current ramp. **(A)** Representative traces showing control, and 0–10 DIV atropine. **(B)** Summary plot with the rheobase for the treatment. **(C)** Shows the summary plot of the interspike interval (ISI). Statistical significance was obtained using an unpaired *t*-test, *p*-values are showed above the graphs.

SUPPLEMENTARY TABLE S1 Summary table showing the values of the excitability parameters of the recorded neurons.

SUPPLEMENTARY TABLE S2 Summary table showing the values of the synaptic transmission of the recorded neurons.
