# Supplementary material for: Long-term muscarinic inhibition increases intrinsic excitability through the upregulation of A-type potassium currents in cortical neurons
Source: Front Cell Dev Biol. 2025 May 27;13:1570424. doi: 10.3389/fcell.2025.1570424 (PMC12149742; doi:10.3389/fcell.2025.1570424)
Supplement: Supplementary file 1 [file DataSheet2.pdf]

Supplementary table 2

EPSC

Chronic	control	Atropine 0-10 DIV	*cohen's d Control-Atrop 0-10 DIV*	*p-value Control-Atrop 0-10 DIV*
**Frequency (Hz)**	5.3 ± 3.4	13.6 ± 7.4	1.783	0.023*
**Amplitude (pA)**	-35.01 ± 10.6	-30.3 ± 16.03	0.366	0.369

Paired t-test

EPSP

Acute	control	Cch	*cohen's d Control-Cch*	*p-value Control-Cch*	Cch-atrop	*cohen's d Control-Cch-Atrop*	*p-value Control-Cch-Atrop*	Cch-wash	*cohen's d Control-Cch-wash*	*p-value Control-Cch-wash*
**Frequency (Hz)**	4.1 ± 1.3	7.7 ± 1.5	-2.746	0.009*	5.9 ± 1.2	-1.343	0.34	6.9 ± 1.3	-2.094	0.047*
**Amplitude (mV)**	2.9 ± 1.2	3.6 ± 1.7	-0.533	0.999	2.7 ± 0.9	0.244	0.999	3.3 ± 0.5	-0.26	0.999

One way ANOVA, Tukey posthoc test

EPSP

Acute	Basal	Atropine	*cohen's d*	*p-value*
**Frequency (Hz)**	3.9 ± 1.4	3.9 ± 1.2	-0.076	0.889
**Amplitude (mV)**	1.7 ± 0.6	1.9 ± 0.8	-0.675	0.27

Paired t-test

EPSP atropine 0-10 DIV, acute Cch

Chronic	Basal	Cch	*cohen's d*	*p value*
**Frequency (Hz)**	8.9 ± 6.7	11.8 ± 9.6	-0.756	0.024*
**Amplitude (mV)**	1.8 ± 1.6	2.03 ± 1.7	-0.365	0.232

Paired t-test
