# Supplementary material for: Long-term muscarinic inhibition increases intrinsic excitability through the upregulation of A-type potassium currents in cortical neurons
Source: Front Cell Dev Biol. 2025 May 27;13:1570424. doi: 10.3389/fcell.2025.1570424 (PMC12149742; doi:10.3389/fcell.2025.1570424)
Supplement: Supplementary file 3 [file DataSheet1.pdf]

Supplementary table 1

Parameter	Control	Atropine 3 h	Control vs Atrop 3 h	Cohen's d	Mecamylamine 3 h	Control vs Mec 3h	Cohen's d	Atropine 0-10 DIV	Control vs atrop 0-10d	Cohen's d	Mecamylamine 0-10 DIV	Control vs Mec 0-10d	Cohen's d
**n**	**12**	**10**			**5**			**11**			**5**		
						Passive properties							
**RMP (mV)**	-70.1 ± 5.4	-65.8 ± 9.3	0.584	-0.63	-62.9 -± 7.1	0.292	-1.06	-68.01 ± 5.4	0.947	-0.31	-67.9 ± 6.8	0.968	-0.34
**Rin (MOhms)**	344 ± 195	258 ± 124	0.855	0.6	270 ± 128	0.999	0.52	315 ± 127	0.999	0.2	189 ± 36	0.982	1.1
**Time contant (ms)**	21.3 ± 6.3	24.6 ± 6.2	0.631	-0.43	21.5 ± 7.9	0.866	-0.03	21.6 ± 8.7	0.988	-0.04	23.6 ± 11.03	0.278	-0.3
						Active properties							
**Rheobase (pA)**	130 ± 33	110 ± 40	0.79	0.5	110 ± 65	0.892	0.5	82 ± 25	0.045*	1.19	100 ± 50	0.642	0.74
**Amplitude (mV)**	92.3 ± 8.6	93.9 ± 11.9	0.99	0.02	88.5 ± 10	0.99	0.53	105.2 ± 10.5	0.15	-1.4	104.2 ± 9.1	0.35	-0.95
**Threshold (mV)**	-28.3 ± 7.6	-28.1 ± 4.1	0.97	-0.88	-24.7 ± 2.7	0.99	-1.6	-35.8 ± 5.2	0.13	0.73	-36.4 ± 7.4	0.16	0.84
**Width (ms)**	2.29 ± 0.56	1.95 ± 0.91	0.69	0.52	2.5 ± 1.25	0.97	-0.24	1.24 ± 0.18	0.022*	1.5	1.37 ± 0.41	0.091	1.3
**Rise time (ms)**	0.89 ± 0.2	0.73 ± 0.23	0.67	0.62	1.02 ± 0.4	0.97	-73	0.59 ± 0.11	0.09	1.23	0.65 ± 0.13	0.41	0.98
**Decay time (ms)**	1.18 ± 0.31	1.04 ± 0.6	0.89	0.39	1.21 ± 0.6	0.99	0.002	0.58 ± 0.12	0.025*	1.5	0.68 ± 0.3	0.13	1.22
**Latency (ms)**	44.6 ± 17.6	31.1 ± 18.9	0.99	0.07	58.1 ± 19.2	0.132	-3.34	33.5 ± 20.6	0.99	0.01	16.9 ± 9.9	0.331	0.42
**Adaptation index**	-0.18	0.01	0.984	0.22	-0.021	0.996	0.2	-0.05	0.932	-0.33	0.07	0.723	0.67
**Post-spike voltage (mV)**	-13.3 ± 10.1	-6.6 ± 14.5	0.348	0.512	-10.7 ± 10.1	0.893	0.257	-22.6 ± 6.9	0.024*	1.075	-10.3 ± 5.8	0.737	0.364

One way ANOVA, Tukey post hoc test
